# Correction: Therapeutic DNA vaccine attenuates itching and allergic inflammation in mice with established biting midge allergy

**DOI:** 10.1371/journal.pone.0311200

**Published:** 2024-09-24

**Authors:** Mey Fann Lee, Yi-Hsing Chen, Pei-Pong Song, Tzu-Mei Lin

The first two authors, Mey Fann Lee and Yi-Hsing Chen, should not have been attributed equal contribution to this work.
