# Supplementary figures and images for: Therapeutic DNA vaccine attenuates itching and allergic inflammation in mice with established biting midge allergy
Source: PLoS One. 2020 Apr 23;15(4):e0232042. doi: 10.1371/journal.pone.0232042 (PMC7179863; doi:10.1371/journal.pone.0232042)

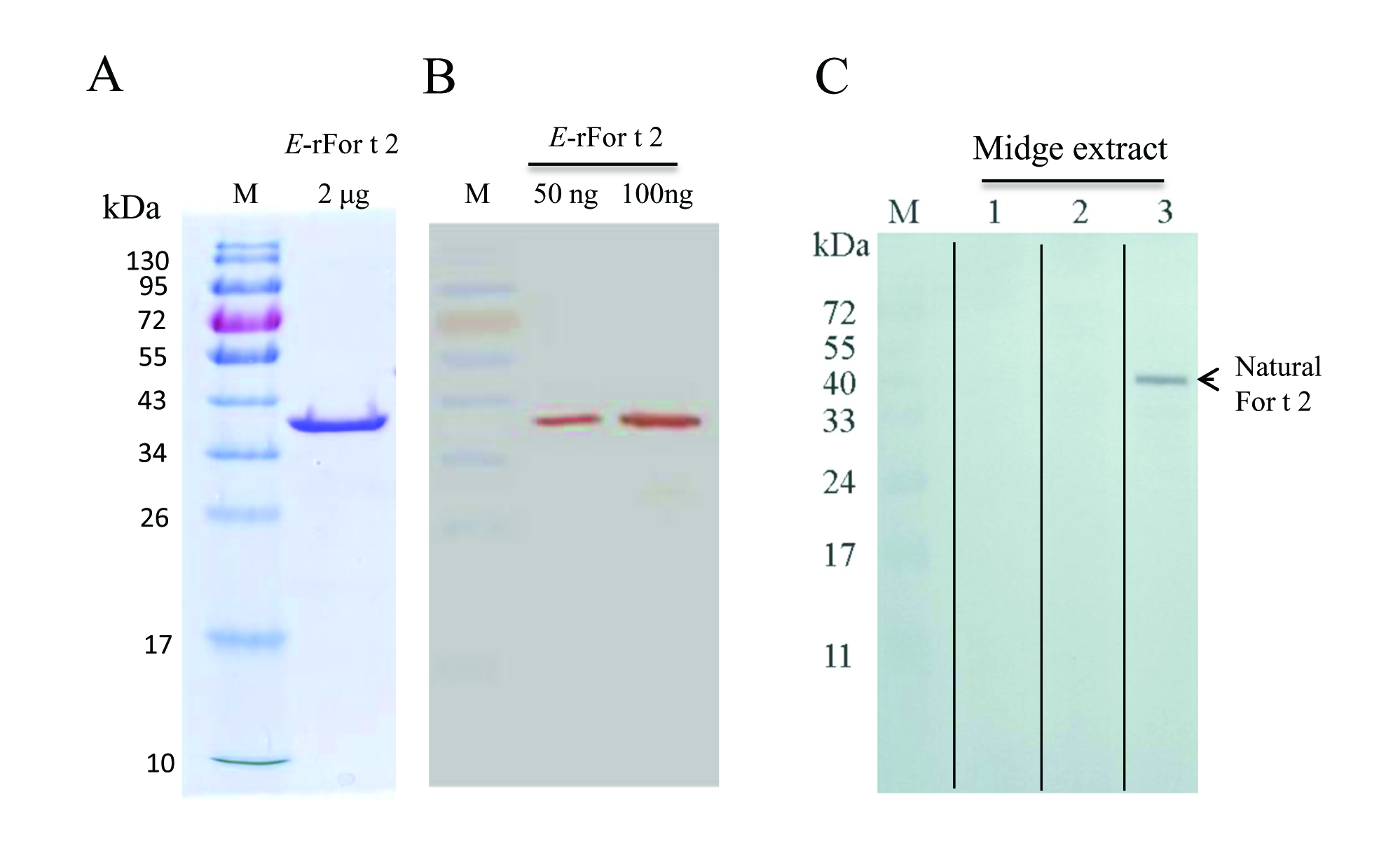

Supplement: S1 Fig — (A) Coomassie Blue-stained SDS-PAGE of purified E.coli-expressed For t 2 recombinant protein. (B) Immunoblotting of E-rFor t 2 or (C) midge extracts with rabbit anti-E-rFor t 2 polyclonal antibodies (lane 3). Lane 1, midge extract probed with non-immunized rabbit serum; lane 2, midge extract probed with pre-immunized rabbit serum. (TIF) [file pone.0232042.s001.tif]
