# Supplementary material for: Therapeutic DNA vaccine attenuates itching and allergic inflammation in mice with established biting midge allergy
Source: PLoS One. 2020 Apr 23;15(4):e0232042. doi: 10.1371/journal.pone.0232042 (PMC7179863; doi:10.1371/journal.pone.0232042)

Original image of Fig 1B captured by the GE Image Scanner III using Epson SilverFast scan software. The cells were cultured for 24 h and then the supernatants from the transfected cells were examined by western blot analysis using rabbit anti-*E*-rFor t 2 polyclonal antibodies. Lane 1, pre-stained protein markers; lane 2, pCI-empty vector; Lane 3, *E.coli*-expressed For t 2 protein as a positive control; lane 4, pCI-For t 2 expressed protein. The reaction was developed using a chemiluminescent substrate and recorded by exposure to an X-ray film.

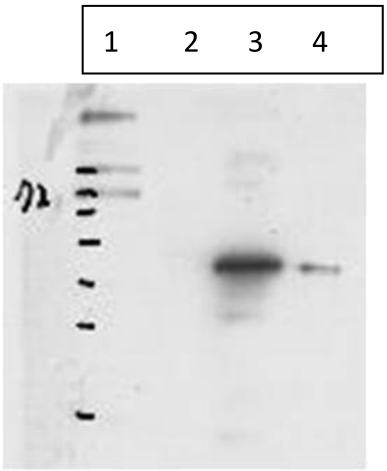

Original image of Fig S1-A. Coomassie blue-stained SDS-PAGE captured by the GE Image Scanner III using Epson SilverFast scan software. Lane 1, Pre-stained protein markers; lane 2, purified *E.coli*-expressed For t 2 recombinant protein.

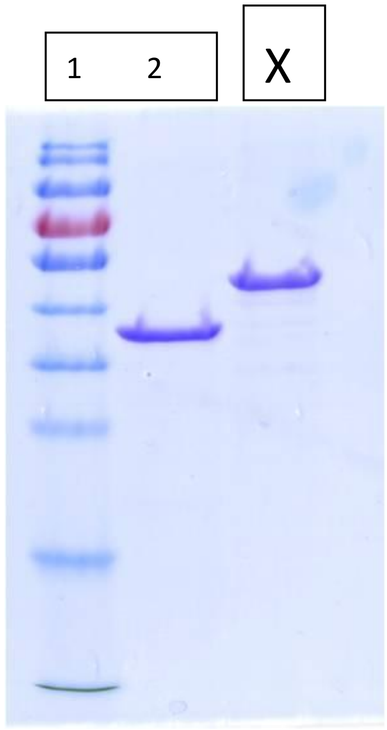

Original image of Fig S1-B shot by Sony NEX-5 digital camera. Lane 1, Pre-stained protein markers; lane 2-3, 50 and 100 ng of *E.coli*-expressed For t 2 recombinant protein immunoblotted by rabbit anti-*E*-rFor t 2 polyclonal antibodies. The membrane was incubated with peroxidase-labeled goat anti-rabbit IgG and visualized using 3-amino-9-ethylcarbazole (AEC) as an enzyme substrate.

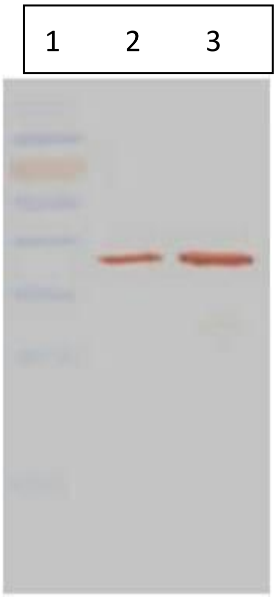

Original image of Fig S1-C shot by Sony NEX-5 digital camera. Lane 1, pre-stained protein markers; lanes 2-4, midge extracts probed with non-immunized rabbit serum, pre-immunized rabbit serum, and rabbit anti-*E*-rFor t 2 polyclonal antibodies, respectively. The membrane was incubated with peroxidase-labeled goat anti-rabbit IgG and visualized using AEC as an enzyme substrate.

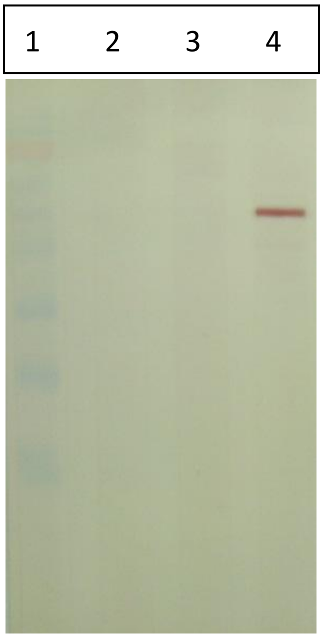

Supplement: S1 Raw images — (PDF) [file pone.0232042.s002.pdf]
